# Prevalence of ulcer forming STIs among HIV-positive women clinic attendees in two Nigerian hospitals

**DOI:** 10.1186/1742-4690-9-S1-P151

**Published:** 2012-05-25

**Authors:** Victoria Awolade, Fajuyi Cantonment, Odogbo Ibadan, Onogbogi Olarewaju, UCH Ibadan

**Affiliations:** 1 Health Initiatives for Safety and Stability in Africa, Ibadan, Nigeria

## Background

The presence of an untreated STI also increases the risk of both acquisition and transmission of HIV by a factor of up to 10. Human Immunodeficiency Virus (HIV) is one of the most common and most dreaded Sexually Transmitted Infection (STI) worldwide. In order to reduce the prevalence of HIV/AIDS infection and subsequently reduce morbidity and mortality among adolescent women, there is need for proper exploration of the relationship between STIs and HIV infection. This study however sought to determine the association between ulcer-forming locally endemic STIs and HIV infection.

## Methods

A seven year retrospective review of 300 case notes of female patients attending the STI clinics of two Nigerian hospitals was done. Those with ulcer-forming sexually transmitted infections were then reviewed to determine age at sexual debut, number of sexual partners and overall sexual behavioural pattern. The results of laboratory tests taken by the patients were also reviewed. Data was analyzed with the use of the SPSS data editor. Chi square tests (95% confidence) were used to determine whether the level of association observed was of statistical significance.

## Results

Fifty-two patients had been treated for ulcerative STIs (17.3%). The mean age at sexual debut was 16.7±1.3years. Thirty - one cases (59.6%) of those confirmed by laboratory tests to have ulcerative STIs were also found to be HIV+. Patients that had 3 sexual partners or more had the highest incidence of STIs and HIV (23.6%). Syphilis was the highest reported ulcerative STI 28.8% followed by Chancroid 21.1%. There is a significant association between ulcer-forming STIs and HIV infection (p=0.01).

## Conclusions

Ulcer producing STIs are associated with HIV infection. An aggressive management of STIs and a more effective contact tracing is needed to reduce new infections of HIV and transmission especially in women.

